# Application of interpersonal psychotherapy for late-life depression in China: A case report

**DOI:** 10.3389/fpsyt.2023.1167982

**Published:** 2023-04-13

**Authors:** Hua Xu, Diana Koszycki

**Affiliations:** ^1^Department of Psychiatry, Shanghai Mental Health Center, Shanghai Jiao Tong University School of Medicine, Shanghai, China; ^2^Emeritus Professor, University of Ottawa, Ottawa, Ontario, Canada; ^3^Institut du Savoir Montfort, Hôpital Montfort, Ottawa, Ontario, Canada

**Keywords:** interpersonal psychotherapy, late-life depression, cultural adaptation, China, intervention

## Abstract

**Objectives:**

Interpersonal psychotherapy (IPT) is an effective treatment for late-life depression, but little is known about its acceptability and efficacy in Chinese patients. This case report describes the use of IPT in a depressed elderly Chinese man.

**Methods:**

The patient was a 79-year-old widower who lives alone in a large city in China. This was his first contact with a mental health specialist. His wife died one ago, and his only child lives in the United States with her husband and children. Due to the COVID-19 pandemic, his daughter could not visit him, and his usual social interactions decreased, leaving him feeling isolated, lonely, and depressed. He was diagnosed with a major depressive episode and initially prescribed venlafaxine. However, he failed to show an adequate response to medication and the side effects were intolerable. He was switched to a low dose of Duloxetine (60 mg) combined with IPT.

**Results:**

The patient’s baseline score on the 17-item Hamilton depression rating scale (HAM-D) was 29, suggesting severe levels of depression. He received 12 sessions of IPT. Role transition was the focus of therapy. Although the patient expressed discomfort in therapy, he developed a good rapport with the therapist and was compliant with treatment. Clinical recovery was achieved at the end of acute IPT treatment (HAM-D score = 1).

**Conclusion:**

Response to IPT was excellent in this elderly patient, but several points should be noted. First, mental health-related stigma in China can affect treatment engagement. Second, older Chinese are reluctant to speak openly about their personal experiences and feelings. Hence, repeated emphasis on the principles of confidentiality in psychotherapy and forming a strong therapeutic alliance are important. Third, the “empty-nest” household is an emergent phenomenon in China. Helping elderly Chinese navigate changes in traditional Chinese living arrangements and negotiate filial piety with offspring who have moved away are important issues to address in therapy.

## Introduction

Late-life depression refers to a depressive disorder that is diagnosed in elderly individuals with no previous history of depression or a recurrence of early onset depression (1, 2). Late-life depression is an increasingly significant public health concern in China with the rising aging population (3). According to studies conducted in the past two decades, around 20–40% of the elderly in China suffered from depressive symptoms (4–7). This may be due in part to the changing face of Chinese society, where out-migration of adult children and the erosion of traditional intergenerational family structure lead to a decline in familiar roles and supports and greater loneliness and isolation (8).

Depression in late life is distinguished from depression among younger ages by its chronicity, high relapse rate, symptoms presentation, and poor response and tolerance to antidepressant medications (9, 10). China has the largest elderly population in the world (11) and as the aging population continues to grow, it is imperative to develop strategies to tackle late-life depression, including early detection and diagnosis, providing better access to affordable mental health services, and increasing social engagement for those affected.

The mainstay treatments for depression include pharmacotherapy and psychotherapy, alone or in combination. Among the psychological therapies, interpersonal psychotherapy (IPT) is a time-limited, evidence-based treatment for depression across the lifespan (12). The therapy is grounded in attachment theory and assumes that irrespective of cause, depression usually occurs within a social and interpersonal context. The goals of IPT are to provide symptomatic relief by helping the patient manage interpersonal stressors that trigger and maintain depressive symptoms, to strengthen and increase social supports, and to improve interpersonal and communication skills (13). The interpersonal problem areas that are the focus of IPT (complicated grief, role disputes, role transitions, and social isolation) fit very well with the interpersonal stressors older persons in China experience in the current changing society, and which are considered important risk factors for late-life depression.

Psychotherapies such as IPT were originally developed in Western countries. In our previous article, we discussed the potential application of IPT for late-life depression in China (14). Although researchers have suggested that the theories and techniques of IPT are universally applicable and that IPT can be applied in different cultures without significant adaptation (13), little is known about its acceptability and potential cultural effect in Chinese elderly patients. These are important questions to address because low treatment acceptability can result in poor treatment engagement and premature drop-out, and culturally-adapted treatment confers better outcome than non-adapted treatment.

In this case report, we discuss how IPT was used to treat depression in an older Chinese man, paying particular attention to traditional Chinese cultural considerations. The patient was treated by the first author (Hua Xu) who is a psychiatrist working in a large psychiatric hospital in Shanghai. She received IPT supervision from the second author (Diana Koszycki), who is a certified IPT supervisor and trainer with the International Society for Interpersonal Psychotherapy.^1^ The patient attended 12 therapy sessions of 50 min in duration. Treatment followed the manual developed by Weissman et al. (13). IPT comprises three phases which are outlined in the manual. In the initial phase (sessions 1–3) the therapist determines if the patient is clinically depressed, explains depression as a medical illness, conducts the interpersonal inventory, and identifies one, or at the most two, interpersonal problem areas that are associated with the patient’s depressive symptoms. The middle phase of IPT (sessions 4–10) focuses on resolving the chosen interpersonal problem area in order to improve mood symptoms. The final phase (sessions 11–12) focuses on consolidating treatment gains, relapse prevention, and assessing the need for further treatment.

## Case report

The patient was a 79-year-old widower who presented to the outpatient unit at a large mental health hospital with the chief complaint of depressed mood for more than 6 months. He had no previous history of mental health problems or contact with a mental health practitioner. The patient was a highly esteemed university professor before he retired from his field. He lived alone in an apartment in Shanghai. His wife died of cancer 2 years ago. They were married for 45 years and had a close relationship. He noted that he was psychologically prepared for her death and experienced typical grief following her death. Therefore, complicated bereavement was ruled out as a focus of therapy.

The patient reports that he began to feel depressed when social distancing strategies and travel restrictions were implemented in Shanghai due to the COVID-19 pandemic. His usual social interactions in Shanghai were markedly diminished, and his daughter and grandchildren, who live in the United States (US), were unable to travel to China to visit him. He felt increasingly lonely, isolated, and depressed. As well, his housekeeper, who came to tidy his apartment daily, took 2 weeks leave, leaving him feeling even more lonely and depressed than before. His depressed mood was accompanied by poor appetite, impaired sleep, fatigue, feelings of weakness, and worry, especially about his daughter’s safety. He was less interested in his usual activities such as calligraphy and Tai-Chi, and he was more withdrawn from friends and family and self-isolating.

The patient obtained a score of 29 on the 17-item Hamilton Depression Rating scale, suggesting severe depression. He was diagnosed with a major depressive episode and initially prescribed venlafaxine. However, he failed to show an adequate response and the side effects of dizziness and constipation were intolerable. He was subsequently switched to Duloxetine (60 mg), but he continued to complain of residual symptoms of depression after a month of treatment. His psychiatrist suggested that his depressive symptoms might be related to social isolation and loneliness due to the COVID-19 restrictions and that he might benefit from IPT. The patient was initially hesitant to start therapy, but with the encouragement of his brother, the patient agreed to work with the psychiatrist for 12 sessions. The patient was provided with psychoeducation about depression and its treatment and socialized to the IPT model.

A review of the patient’s current interpersonal network revealed that he had a fairly good support system for someone his age. His daughter is his only child and she lives in the US with a man who is also originally from China; they have three children. He described a good relationship with his daughter and grandchildren and they spoke regularly *via* WeChat. He had a close relationship with his deceased wife’s brother and his family, who also lived in Shanghai. His sister, who ordinarily lives in the US, was visiting Shanghai when the pandemic began. She had to extend her stay in China because of international travel restrictions. She was an essential source of support to the patient during her extended stay in Shanghai. He also had a brother who lives in the US and they spoke often. Additionally, he had several long-time friends and colleagues who live in Shanghai. They used to meet regularly and traveled together before the pandemic.

After reviewing the interpersonal and social context of the patient’s depression the psychiatrist formulated the case as a role transition, which involved difficulty the patient has had adjusting to the changes in his usual activities and social interactions brought about by the pandemic. The patient readily accepted this IPT formulation and treatment goals were collaborative set and included helping the patient resume usual interactions with his group of friends and colleagues, increase his level of support from family members, and re-engage in activities he found enjoyable. His daughter, the most important person to him, called him almost every day *via* Wechat to support him. She was aware that he was receiving treatment for depression and was very concerned about his well-being. During one of his therapy sessions, the patient reported that his daughter wanted to make him feel less lonely, and arranged for his wife’s old friends to travel to his apartment for a surprise visit; they brought food and talked to each other for the entire day. In another session, the patient mentioned that he arranged to visit his brother-in-law’s home and was delighted to hear many stories about his wife’s family. He was surprised that he never knew about these stories. The psychiatrist highlighted how these positive social interactions had a beneficial impact on his mood and emphasized how staying connected to others was important for his psychological well-being.

While discussing his daughter, the patient revealed that he felt sad and lonely that his only child did not live near him, and these feelings were intensified by the pandemic and social distancing requirements. The patient did not want to move to the US to live with his daughter because he felt he would be unhappy in an unfamiliar cultural context and he preferred living in Shanghai. He was proud of his daughter and described her as an intelligent and capable person and good mother. He admitted that he disapproved of his daughter’s choice of husband, and he resented his son-in-law for separating him from his daughter and grandchildren. He felt left behind and missed the close emotional ties and support a traditional intergenerational Chinese household can provide. As well, he felt regret that his daughter gave up a promising career to marry someone who did not meet his expectations and become a stay-at-home mother. The psychiatrist helped the patient explore his negative feelings towards his son-in-law with the goal of helping him develop a more balanced perspective of him. Eventually, the patient was able to acknowledge that his son-in-law was a hardworking and responsible man who moved with his daughter to the US for a better life. As well, the patient became more accepting of his daughter’s life choices and he was able to admit that she was happy in her life.

At the beginning of therapy, the patient had some difficulties expressing his thoughts and emotions, but as the treatment progressed, he learned to express his complicated feelings about his relationships and his circumstances. Although he was in relatively good health, he began to discuss his fear of declining health and concern about not having someone around in the event of a medical emergency. To address this fear, the psychiatrist helped him explore options to increase his sense of safety living alone, such as asking family, friends and neighbors to regularly check in on him and identifying people who could care for him if he suddenly fell ill. As therapy progressed, he opened up more and discussed his worst fear, which saddened him greatly. He was afraid of dying alone at home and worried about who would take care of his body in a timely manner when dead. In traditional Chinese culture, rules around death are important. Adult children are not only obligated to take care of their aging and frail parents, but they are also responsible for funeral planning immediately after their death. The psychiatrist encouraged the patient to talk with his daughter about this concern, and to identify an extended family member who could arrange his funeral in the event his daughter could not fulfill this filial duty, even though this would be a significant departure from the traditional Chinese custom.

Despite the patient’s discomfort expressing his complicated feelings and discussing painful topics, his mood gradually improved with therapy. During the termination phase of therapy, treatment gains were reviewed and the patient acknowledged that being socially connected contributed to his improved mood. He had more positive feelings towards his son-in-law, and attributed this to developing a more balanced perspective about him as well as his daughter’s life in the US. He also admitted that exploring potential supports from people around him and asking for help relieved his worries about illness and death. He demonstrated an excellent response to IPT and at the end of the 12 therapy sessions, he obtained a HAM-D score of 1.

After acute IPT treatment, the patient was followed every 3 months for 2 years. At the last follow-up appointment his mood continued to be euthymic. He no longer required hypnotics and the dose of Duloxetine was reduced to 20 mg. The patient reported that he continued to implement what he learned in therapy. He had resumed his regular activities and was socially active. He began to travel again with his close friends, and they discussed the possibility of living together so they could support each other through health challenges and reduce loneliness. His mood has been stable for 2 years without fluctuations.

## Discussion

The use of psychotherapy as a treatment option for psychological disorders is rising in China. For example, psychodynamic, psychoanalytic, and cognitive behavioral therapies have received considerable attention since the 1990s (15). IPT is relatively new in China and efforts to disseminate this therapy into routine clinical settings is currently underway (16). Despite these important efforts, research on the use of IPT in China is limited, and little is known about how to deliver culturally sensitive IPT for depression across the life-span.

This case suggests that IPT is a well-accepted and potentially efficacious treatment for late-life depression in China. Prior to receiving IPT, the patient was prescribed Duloxetine with a dose of 60 mg. After 1 month of treatment, he continued to experience residual depressive symptoms with anxiety. During the 12 IPT sessions, the dose of Duloxetine was maintained at 60 mg, and the patient finally achieved clinical recovery with a HAMD score of 1. The patient described in this report was able to form a good therapeutic alliance with the psychiatrist and he demonstrated an excellent response to acute IPT. Four interpersonal problem areas are the foci in IPT: complicated grief, role transitions, role disputes, and interpersonal deficits/sensitivity. For this patient, the interpersonal problem that was linked with the onset of depression was role transition. As noted earlier, the problem areas that are the central focus of IPT are universal and the strategies and techniques of this therapy can be easily applied to individuals across various cultural backgrounds (13, 17). However, traditional Chinese culture plays a significant role in the healing process (18), and understanding the essence of the structure of Chinese thought and beliefs can enhance the relevance, meaningfulness, and effectiveness of IPT (17).

There are a number of cultural issues to consider when using IPT with an elderly Chinese patient. The first is mental illness-related stigma and poor mental health literacy. Stigma and discrimination towards people with mental illness are widespread in China (19, 20). A large proportion of Chinese hold stigmatizing attitudes towards people with depression (21) including views that depressed people are unpredictable and dangerous (22). As well, mental health literacy tends to be low among older Chinese adults, making it more likely for them to endorse negative cultural beliefs about mental illness (23) and develop less positive attitudes towards seeking mental health care (24). These negative views can create barriers to help-seeking and treatment compliance.

In this case, the patient believed there was a stigma associated with seeking treatment from a psychiatrist and a psychotherapist. Fortunately, the patient’s brother encouraged him to try therapy and suggested that he would likely benefit from it. Although Chinese people are less inclined to discuss mental health difficulties with friends and family because of shame, embarrassment, and a desire not to burden others, this case suggests that family members with positive attitudes towards mental health treatment can play a key role in encouraging elderly Chinese to comply with recommended psychosocial treatments (25–27). Psychoeducation is also an important component of IPT and acquiring basic knowledge of depression and its treatment can help minimize the impact of unhelpful cultural attitudes about mental illness and its treatment held by patients and their families.

A second issue concerns the expression of affect. IPT is an affect-focused intervention and helping patients acknowledge, accept, and manage painful feelings is a key therapeutic technique. Talking about feelings was clearly a challenge for this patient. This is not uncommon as elderly Chinese often have difficulty identifying feelings, feel reluctant to speak openly about their difficulties with a stranger, even a professional, and tend to express their emotional pain physically, perhaps because somatization allows people to be ill without stigma (15); these factors can potentially reduce their responsiveness to IPT (28). Another factor that could account for discomfort elderly Chinese might have about disclosing painful feelings with a therapist is suspicion of the principles of patient-therapist confidentiality. It took more time to discuss the principles of confidentiality in therapy with this patient than it typically takes with younger Chinese patients. To develop a “sense of trust in doubt” (29) in elderly depressed Chinese patients, the IPT therapist must carefully attend to the formation and maintenance of the therapeutic alliance and sensitively address concerns about privacy and confidentiality so the patient can feel safe to self-disclose in therapy. As the therapeutic bond develops, the therapist should gently encourage the elderly patient to discuss their complicated feelings within a supportive therapeutic relationship, reframe their somatic symptoms of depression into culturally relevant psychological constructs, help them find appropriate ways to communicate their needs and feelings with significant others, and increase their comfort in seeking out the support of others (14), especially when they feel distressed.

Third, views of sickness and death in China are very much influenced by traditional culture. Many Chinese people avoid discussing death-related concerns openly because talking about death or serious physical illness is a taboo subject and is believed to bring bad luck (26). When an elderly Chinese person falls ill or dies, it is customary for their adult children to take care of them and arrange their funeral after death. This is an important aspect of filial piety. The geographic distance between the patient and his daughter was a critical stressor that contributed, in part, to his depressed mood, but also to his preoccupation with dying alone and his funeral arrangements. It is often difficult for elderly Chinese to seek assistance or discuss death arrangements with others, including their children. For some elderly Chinese, this is often a stressor that can contribute to or exacerbate depressed mood. In this clinical example, the IPT therapist was able to decrease the patient’s preoccupation with dying at home alone and his funeral arrangements by helping him explore his feelings about this sensitive topic and identify potential supports if his daughter was unable to return to China without delay to arrange his funeral. Understanding the additional meaning from traditional Chinese culture about sickness and death may help the IPT therapist find more individual, balanced, and adapted ways of working with depressed elderly patients who express these types of concerns.

Finally, because of the one-child policy established in China in 1979 and increased out-migration of young adults in modern Chinese society, family support systems for the elderly are weakening (30, 31). The elderly patient, in this case, is just one of the many “empty nesters,” and this is not rare. A recent meta-analysis showed that the prevalence of depression among empty-nest elderly is high, with a pooled prevalence rate of 38.6% (32). This patient valued the concept of family loyalty, which places the elderly parent in the center rather than the periphery of the nuclear family. Even though this patient had strong social ties in Shanghai, the physical distance between him and his daughter and grandchildren caused him much pain, and he harbored feelings of resentment towards his son-in-law for relocating his family to the US. In working with empty-nest elderly Chinese, the IPT therapists needs to be sensitive to important role losses these individuals experience, help them better adjust to having a long-distant relationship with their adult child and grandchildren, explore expectations for filial piety at a distance, and encourage them to increase social interactions with local extended family.

To our knowledge this is the first case report that describes the application of IPT in an elderly Chinese patient. The patient demonstrated a good response to IPT and his HAMD score decreased from an initial score of 29 to 1. While this case report described some relevant culturation adaptations of IPT in an elderly Chinese patients, additional case studies are needed to further describe culturally sensitive IPT in the treatment of late life depression in China.

## Conclusion

In conclusion, 12 sessions of IPT was well accepted, tolerated, and efficacious for the treatment of depression in an elderly patient treated in an outpatient mental health facility in a large urban city in China. The benefits of IPT were enhanced by addressing the patient’s cultural beliefs, values, and practices. Stigma, the expressive characteristics of older Chinese, and the importance of filial piety are important elements that should be considered when applying IPT for the treatment of late-life depression in China. As well, it is necessary to use language that is in line with the age and culture of the patient, to gently encourage the patient to talk openly about feelings, and to help them find appropriate ways to improve and strengthen important family and social ties, especially ties with adult children who live abroad.

## Data availability statement

The original contributions presented in the study are included in the article/supplementary material, further inquiries can be directed to the corresponding authors.

## Ethics statement

The studies involving human participants were reviewed and approved by the Ethics Committee of Shanghai Mental Health Center. The patients/participants provided their written informed consent to participate in this study. Written informed consent was obtained from the individual(s) for the publication of any potentially identifiable images or data included in this article.

## Author contributions

HX drafted the manuscript. DK supervised HX and made critical revisions to the manuscript for important intellectual content. All authors made substantial contributions to the conception and design, drafted the article or revised it critically for important intellectual content, gave final approval of the version to be published, and agreed to be accountable for all aspects of the work. All authors contributed to the article and approved the submitted version.

## Funding

This work was supported by the Ministry of Science and Technology (2022YFC3600600), the Science and Technology Commission of Shanghai Municipality (18411961500), the Shanghai Health Committee (202040366), and the Shanghai Mental Health Center (grant number 2017-JY-12).

## Conflict of interest

The authors declare that the research was conducted in the absence of any commercial or financial relationships that could be construed as a potential conflict of interest.

## Publisher’s note

All claims expressed in this article are solely those of the authors and do not necessarily represent those of their affiliated organizations, or those of the publisher, the editors and the reviewers. Any product that may be evaluated in this article, or claim that may be made by its manufacturer, is not guaranteed or endorsed by the publisher.

## Footnotes

^1^ https://interpersonalpsychotherapy.org
